# Building bridges with zebrafish: highlights of the ‘Zebrafish Models in Translational Medicine’ meeting

**DOI:** 10.1242/dmm.014571

**Published:** 2013-11

## Abstract

Inspired by the growing prominence of the zebrafish model in biomedical research, EuFishBioMed (www.eufishbiomed.kit.edu/) and ZF-HEALTH (zf-health.org/) recently joined forces to organise a meeting with the primary aim of forging collaborations between the established ‘zebrafish community’ and newcomers that hope the model will shed light on unanswered questions in their disease area. The 3-day meeting, ‘Zebrafish Models in Translational Medicine’, which was co-sponsored by *Disease Models & Mechanisms* (DMM), was held in a small town in the outskirts of Paris (Gif-sur-Yvette). With no more than 100 participants, opportunities for networking were plentiful.

**Figure:**
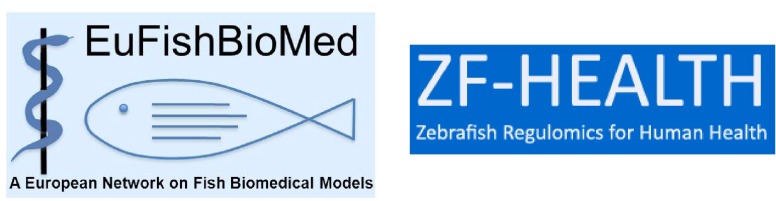


The meeting brought together a mixed bag of researchers, with sessions divided into three key disease areas: neurodevelopmental and psychiatric disorders, cancer, and metabolic diseases. Most of the invited speakers have never worked on zebrafish. Scientific coorganiser Laure Bally-Cuif, CNRS, explained that these speakers were selected to highlight the challenges and opportunities in a specific medical area in order to stimulate cross-discipline debate on current models and approaches.

In the opening plenary lecture, Jesus Benavides, former director of CNS drug discovery at Sanofi, provided his perspectives on the ultimate challenge in medicine – the translation of scientific understanding to therapeutic innovation. Using neurodegenerative disease as an example, Jesus emphasised the lengthy timeframes, significant costs and discouragingly high attrition rates associated with drug development. He presented the audience with a somewhat gloomy outlook: despite advances in our understanding of drug toxicity, lack of drug efficacy remains a major problem. This has been largely attributed to the poor predictive potential of preclinical animal models, and Jesus argued that the problem lies in the fact that models don’t truly recapitulate the pathophysiology of the disease they are intended to model. He proposed that building up models based on disease endophenotypes (rather than attempting to mimic a whole disease) could provide more translatable findings, and that biomarker discovery and validation is an important step in pharmaceutical research.

The core sessions kicked off with a talk by Stephane Jamain, Inserm, on the genetics of bipolar disorder. Stephane described how, despite the genetic complexity of the condition, collaborative genome-wide association studies (GWAS) have provided a number of candidate single-nucleotide polymorphisms (SNPs) for follow-up analysis. His group focuses on phenotypic refinement of pathways underlying the common symptoms, including abnormalities in sleep/wake cycles. They have shown that rare, deleterious variants in *ASMT*, which is involved in the melatonin biosynthesis pathway, contribute to bipolar disorder susceptibility. The majority of insights into bipolar disorder and overlapping psychiatric diseases have been gained through human genetics studies and *in vitro* analyses, in part because of the lack of suitable animal models. Given the increasing understanding of its neurobehavioural characteristics, the zebrafish model might meet this need, both for functional characterisation of mutations and drug screening.

**Figure:**
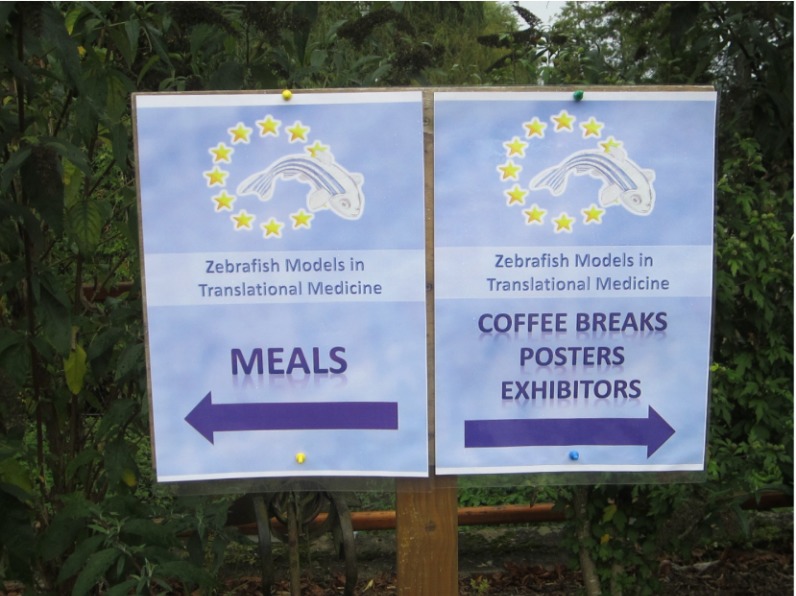


Maria Karayiorgou, Columbia University, gave a talk on another psychiatric disorder that is associated with a high degree of clinical heterogeneity: schizophrenia. Schizophrenia is unusual because, despite being associated with reduced reproductive success, it is fairly common in the population. Maria explained how genetic analyses have provided a plausible explanation for this, by showing that *de novo* mutations continually replenish highly penetrant risk variants for schizophrenia. Recent exome sequencing studies have revealed a host of rare *de novo* mutations. Because current therapies manage the symptoms but don’t provide a cure, research efforts are now concerned with prioritising molecular targets for drug development. Using mouse models, Maria’s group recently pinpointed a microRNA involved in the pathophysiology of schizophrenia associated with 22q11.2 microdeletions.

In one of the zebrafish-focused talks of the psychiatric disorders session, Camila Esguerra described how her group at KU Leuven has developed genetic and pharmacologically induced zebrafish models of seizure, and is using these to identify anticonvulsant compounds and explore mechanisms of treatment-resistant epilepsy. Some of the promising compounds have been cross-validated in rodent models, underlining how zebrafish can be used as a complementary model in large-scale drug discovery.

Michael Brand, TU Dresden, discussed his research based on the astounding ability of the adult zebrafish brain to regenerate. He explained that brain regeneration is an important process to study from a translational point of view, because the basic processes can provide insight into neurodegenerative disease and also traumatic brain injury (TBI), which remains a relatively understudied major health problem. By applying brain lesion assays in zebrafish, his group has shown that inflammation plays an essential role in regeneration. They now aim to explore this phenomenon in mammalian models, which have already provided a wealth of insights into TBI.

Marina Mione, KIT, one of the scientific organisers of the meeting, presented her recent, unpublished findings into tumours triggered by the oncogene *Ras*. Marina, who has worked on zebrafish for many years, analysed global microRNA expression in three different zebrafish tumour models, revealing a suite of microRNAs that are consistently upregulated. Downstream targets of the microRNAs were determined, providing insight into the pathways involved in Ras-induced oncogenesis from zebrafish to humans.

Several discussions highlighted the advances made in uncovering oncogenic mechanisms using zebrafish. However, mice also remain at the forefront of cancer studies, as demonstrated in a talk by Jean Soulier, University Paris Diderot, on leukaemia. Relapse following treatment is discouragingly common in leukaemia, and relapsed patients often respond poorly to treatment, so dissecting clonal evolution is an important step towards improving survival rates. Jean’s group models clonal evolution in leukaemia by xenografting patient cells into immunodeficient mice. In a recent study, genomic profiling tools were used to compare T-ALL patient samples at the time of diagnosis and after engraftment into mice. These analyses showed that xenografts display a gene expression signature and pattern of new genetic lesions that are reminiscent of those in relapse patient samples. Xenograft cells were also found to be less sensitive to drugs, demonstrating that the approach can be used to establish a robust model of the process of relapse in leukaemia.

Rounding up the meeting, the metabolic disease session included talks on hypertension, diabetes, hypoxia and vascular diseases. Patricia Munroe, QMUL, gave an overview of the number and types of genetic variants associated with hypertension, as revealed by collaborative GWAS and elegant large-scale candidate studies. Now, Patricia and other lead researchers in cardiovascular genomics are eagerly awaiting the generation of knockout models via the IMPC pipeline (www.mousephenotype.org/) to help them to assign functions to the many loci identified. Work by the Müller group in collaboration with Atilla Sik (University of Birmingham, UK) convincingly demonstrates that zebrafish embryos can be used for downstream drug screening for cardiac diseases: they recently described an approach to detect drug-induced cardiac dysfunction in zebrafish using electrocardiography. Compared with mammalian models, using zebrafish to assess cardiac drug toxicity is less costly and potentially faster.

**Figure:**
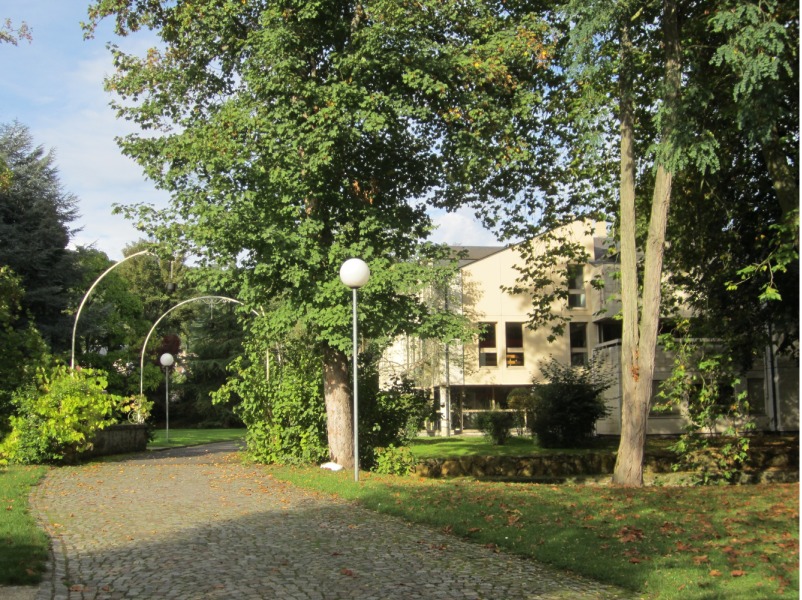


Coming full circle, Peter Ratcliffe (University of Oxford, UK), who discussed the therapeutic potential of HIF hydroxylase inhibitors for anaemia and ischaemic vascular diseases, emphasised that studies in animal models don’t always translate to viable drug targets. He believes that zebrafish models of disease will play a key role in target discovery for a range of diseases.

As demonstrated at this meeting, the applications of zebrafish are abundant and far-reaching, and the opportunities to share the unique tools and approaches with those outside the fish community are continually growing. In support of this, DMM is compiling a Special Issue with the aim of showcasing zebrafish-based translational research, including research that utilises zebrafish together with other models. To find out more, visit the special issue page at: http://dmm.biologists.org/site//misc/zebrafish.xhtml.

